# Multimodality imaging of Abernethy malformation

**DOI:** 10.1111/echo.15324

**Published:** 2022-02-16

**Authors:** Ahmed Y. El‐Medany, Gui Rego, Matthew Williams, Stephen Lyen, Mark Turner

**Affiliations:** ^1^ Department of Cardiology Bristol Heart Institute Bristol UK; ^2^ Department of Radiology Bristol Royal Infirmary Bristol UK

**Keywords:** Abernethy malformation, CT, echocardiography, MRI, portal vein

## Abstract

Abernethy malformation, or congenital extrahepatic portosystemic venous shunt, is a rare anomaly involving the portal venous system. Despite its rarity, it is increasingly being reported, and therefore, it is important to diagnose given the potential adverse clinical consequences if left untreated. It has a spectrum of presentations, ranging from complete lack of symptoms, to causing hepatic carcinoma, hepatic encephalopathy, severe pulmonary hypertension, and diffuse pulmonary arteriovenous malformation. We herein describe the case and echocardiographic, computed tomography, and magnetic resonance imaging findings of a transgender individual, with this anomaly detected incidentally during adulthood.

## 1 CASE PRESENTATION

**FIGURE 1:**
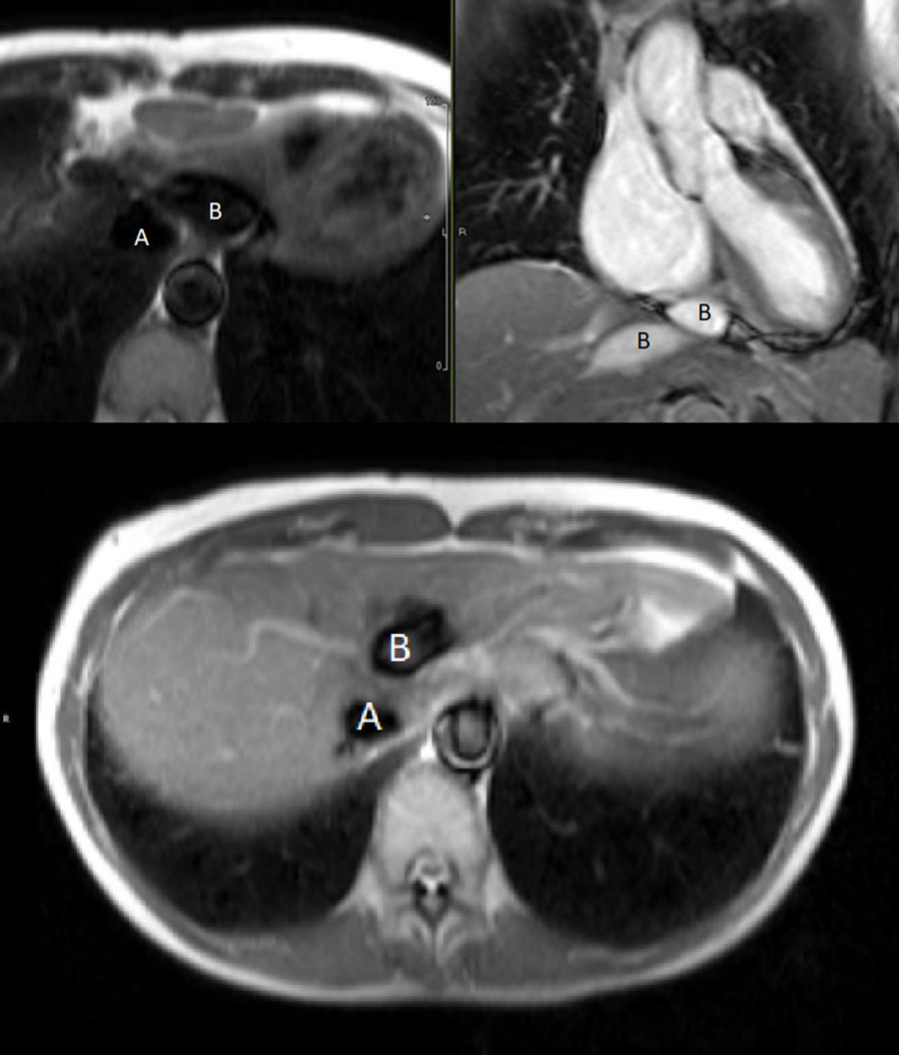
Magnetic resonance images of incidental Abernethy malformation. Axial half Fourier single‐shot turbo spin‐echo (top‐left and bottom) and coronal fast imaging with steady‐state precession (top right) images demonstrating the inferior vena cava (A) and extrahepatic portosystemic shunt (B)

**FIGURE 2:**
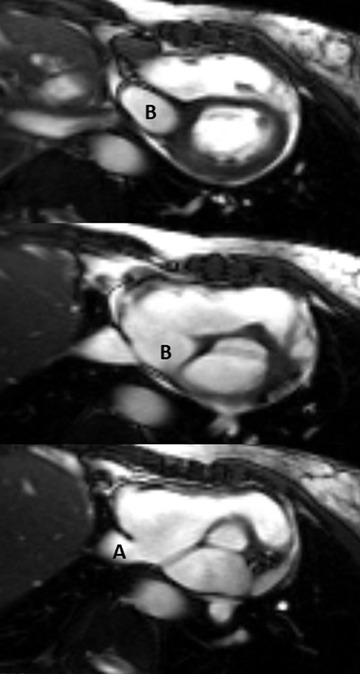
Magnetic resonance images of incidental Abernethy malformation. Short axis steady‐state free precession cine stack demonstrating the abnormal portal vein (B) and the inferior vena cava (A) entering the right atrium

**FIGURE 3:**
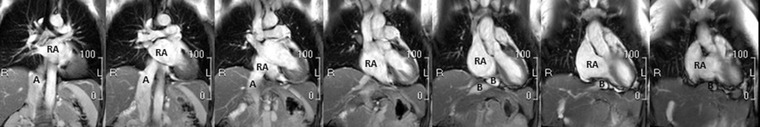
Magnetic resonance images of incidental Abernethy malformation. Coronal fast imaging with steady‐state free precession, posterior to anterior stack, demonstrating the inferior vena cava (A) and extrahepatic portal vein (B) draining into the right atrium (RA)

**FIGURE 4:**
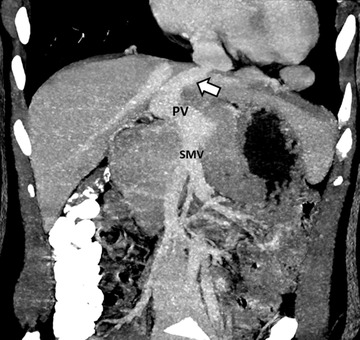
Coronal maximum intensity projection contrast‐enhanced computed tomography of the upper abdomen in the portal venous phase demonstrating extrahepatic portosystemic shunt (arrow), portal vein (PV), and superior mesenteric vein (SMV)

A 41‐year‐old transgender female presented to the cardiology clinic with long‐standing symptoms of frequent palpitation and occasional dizziness. Previous echocardiography 2 years prior reported on a globular left ventricle and mild left atrial dilatation, although mentioned no other abnormalities. She had had previous equivocal Lyme disease serology consistent with previous deer bites, and therefore a cardiac magnetic image resonance (CMRI) scan was organized to assess for possible myocarditis. This investigation revealed a large anomalous porto‐sytemic shunt in keeping with Type 1b Abernethy malformation (Figures 1, 2, 3). She was subsequently referred to the adult congenital heart disease (ACHD) team and the Hepatologists with regards to further investigation and consideration of percutaneous shunt occlusion. A critical flicker frequency test was organized to assess for possible subtle hepatic encephalopathy in the context of a large porto‐systemic shunt, which was ultimately reassuring. Computed tomography (CT) confirmed a large aberrant vessel extending from the portal vein to the right atrium (Figure 4). No significant hepatic lesion was identified. In the absence of symptoms, or obvious benefit to undertaking an occlusion of this lady's abnormal venous connection, the decision was made to avoid percutaneous shunt occlusion.

**FIGURE 5:**
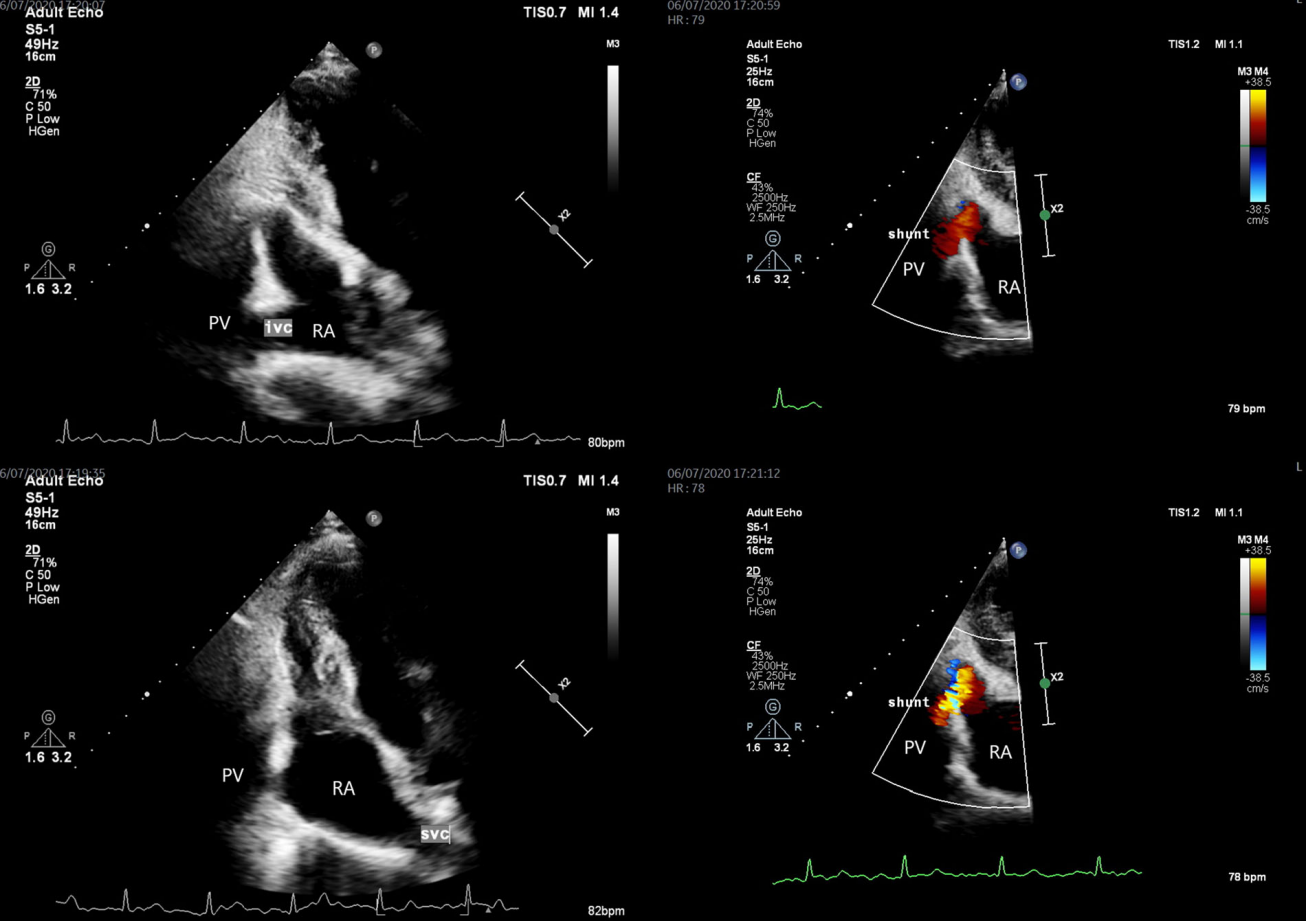
Subcostal transthoracic echocardiography imaging demonstrating the inferior vena cava (ivc), superior vena cava (svc), and extrahepatic portosystemic shunt (shunt) – Color doppler demonstrates venous flow diversion from the portal vein (PV) into the right atrium (top and bottom right)

Follow‐up echocardiography 12 months later (Figure 5) effectively demonstrated an abnormal connection between the portal system and right atrium. Color doppler demonstrated venous flow diversion from the portal vein into the inferior vena cava (Figure 5 and Supplementary Material).

## 2 DISCUSSION

Abernethy malformation is a congenital extrahepatic portosystemic shunt (CEPS), whereby most of the intestinal and splenic venous blood bypasses the portal vein and drains directly into the inferior vena cava (IVC) through abnormal communication. Type 1 CEPS is characterized by the absence of intrahepatic portal vein branches and an end‐to‐side portocaval shunt, whereas in type 2 CEPS the intrahepatic veins are hypoplastic but patent, and a side‐to side shunt diverts blood from the portal vein to the IVC. Type 1 CEPS can be further classified into type 1a, when the superior mesenteric and splenic veins drain separately into the IVC, and type Ib when these veins form a common anastomosis before draining into the IVC.^1^, ^2^ This case is an example of type 2 CEPS.

Abernethy malformation is diagnosed via noninvasive cross‐sectional imaging such as transthoracic echocardiography, CT, or magnetic resonance imaging (MRI), which demonstrates the shunt and any intrahepatic portal vein branches.^3^

To date, less than 300 cases of CEPS have been reported. The spectrum of clinical presentation ranges from completely asymptomatic forms to severe forms of hepatic encephalopathy (HE), hepatopulmonary syndrome (HPS), and pulmonary arterial hypertension (PaHT).^4^, ^5^, ^6^, ^7^, ^8^ Nodular liver lesions are frequently identified, although most of these nodules are benign. However, hepatocellular carcinomas (HCCs) and adenomas, among other neoplastic lesions, have been reported.^3^, ^8^ Furthermore, CEPS is commonly associated with congenital cardiovascular abnormalities, such as atrial and ventricular septal defects, patent foramen ovale, patent ductus arteriosus, and tetralogy of Fallot.^4^, ^7^

Shunt closure is considered in symptomatic patients and as a prophylactic treatment early in the course of the disease to prevent the development of severe complications such as HE. Despite shunt closure, the risk of complications never completely disappears, and thus regular surveillance for HCC is important.^4^

## Supporting information

Supplementary Information
